# TRIM26 Induces Ferroptosis to Inhibit Hepatic Stellate Cell Activation and Mitigate Liver Fibrosis Through Mediating SLC7A11 Ubiquitination

**DOI:** 10.3389/fcell.2021.644901

**Published:** 2021-03-25

**Authors:** Yiming Zhu, Chihao Zhang, Mingzhe Huang, Jiayun Lin, Xiao Fan, Tao Ni

**Affiliations:** ^1^Department of General Surgery, Shanghai Ninth People’s Hospital, School of Medicine, Shanghai Jiao Tong University, Shanghai, China; ^2^Department of Plastic and Reconstructive Surgery, Shanghai Ninth People’s Hospital, School of Medicine, Shanghai Jiao Tong University, Shanghai, China

**Keywords:** liver fibrosis, ferroptosis, hepatic stellate cell, TRIM26, SLC7A11

## Abstract

Hepatic stellate cells (HSCs) are activated by inflammatory mediators to secrete extracellular matrix for collagen deposition, leading to liver fibrosis. Ferroptosis is iron- and lipid hydroperoxide-dependent programmed cell death, which has recently been targeted for inhibiting liver fibrogenic processes. Tripartite motif-containing protein 26 (TRIM26) is an E3 ubiquitin ligase that functions as a tumor suppressor in hepatocellular carcinoma, while little is known about its function in liver fibrosis. In the present study, the differential expression of TRIM26 in normal and fibrotic liver tissues was examined based on both online databases and specimens collected from patient cohort. The effects of TRIM26 on HSCs ferroptosis were examined *in vitro* through evaluating cell proliferation, lipid peroxidation, and expression of key ferroptosis-related factors. *In vivo* function of TRIM26 in liver fibrosis was examined based on CCl_4_-induced mice model. We found that TRIM26 was downregulated in fibrotic liver tissues. The overexpression of *TRIM26* inhibited HSCs proliferation, promoted lipid peroxidation, manipulated ferroptosis-related factor expressions, and counteracted the effect of iron inhibitor deferoxamine. Moreover, TRIM26 physically interacted with solute carrier family-7 member-11 (SLC7A11), a critical protein for lipid reactive oxygen species (ROS) scavenging, and mediated its ubiquitination. In addition, *TRIM26* overexpression induced HSCs ferroptosis and mitigated CCl_4_-induced liver fibrosis in mice. In conclusion, TRIM26 promotes HSCs ferroptosis to suppress liver fibrosis through mediating the ubiquitination of SLC7A11. The TRIM26-targeted SLC7A11 suppression can be a novel therapeutic strategy for liver fibrosis.

## Introduction

Liver fibrosis is characterized by the activation of hepatic stellate cells (HSCs) and excessive deposition of fibrillary collagen (Tomita et al., 2014; Tacke and Trautwein, 2015). It is an inflammatory and fibrogenic process, during which massive inflammatory mediators activate HSCs for subsequence differentiation into myofibroblasts (Puche et al., 2013; Li et al., 2015). The activated HSCs continuously secrete extracellular matrix that contains abundant collagen I and III, resulting in collagen accumulation and fibrotic scarring (Schuppan, 2015). Hepatic fibrosis can be induced by viral infections, excessive alcohol consumption, drug overuse, aberrant metabolic activities, or autoimmunity (Zhang et al., 2016). The abnormal synthesis of inflammatory cytokines and accumulation of reactive oxygen species (ROS) gradually compromise the normal hepatic structure and functions, which ultimately progresses to liver cirrhosis, hepatocellular carcinoma, and liver failure (de Souza-Cruz et al., 2016). At present, available treatments for liver fibrosis are either preventive or temporarily effective (Weiskirchen and Tacke, 2016; Zhang et al., 2016), which highlights the necessity to identify novel molecular targets for developing efficient, long-lasting therapeutic strategies.

Ferroptosis is a recently discovered form of programmed cell death, which relies on iron-driven lipid peroxidation (i.e., accumulation of lipid ROS) (Dixon et al., 2012; Gaschler and Stockwell, 2017). It plays a crucial role in the pathological processes of multiple organs, including brain, heart, kidney, and liver (Xie et al., 2016). Specifically, iron overload in liver not only has been recognized to trigger liver injuries, but also markedly influences liver fibrosis (Bloomer and Brown, 2019; Macias-Rodriguez et al., 2020). Since activated HSCs serve as the primary mediator of the hepatic fibrogenic process (Tsuchida and Friedman, 2017), novel therapeutic strategies for liver fibrosis can be established through inducing the ferroptosis of these activated HSCs. Furthermore, the solute carrier family-7 member-11 (SLC7A11) subunit in cystine/glutamate antiporter system is responsible for exporting intracellular glutamate and internalization of extracellular cystine, generating glutathione (GSH) for lipid ROS scavenging (Sato et al., 1999; Dixon et al., 2014; Yang et al., 2014). It was previously reported that inhibiting SLC7A11 in HSCs could induce the ferroptosis of myofibroblasts and attenuate liver fibrosis (Xie et al., 2016). Therefore, SLC7A11 suppression possesses potential therapeutic functions in preventing or treating hepatic fibrosis.

Tripartite motif-containing protein 26 (TRIM26) is a member of TIRM family. A similar characteristic structure, which includes a RING-finger domain, one or two B-boxes, and a coiled coil domain, has been observed in TRIM proteins. Many members of this family function as E3 ubiquitin ligases and are involved in a wide range of biological processes, and their abnormal expression leads to a variety of pathological conditions, such as inflammation, viral infection, and cancer (Hatakeyama, 2017; van Gent et al., 2018). Recent reports have reported the E3 ubiquitin-ligase activity of TRIM26 (Wang P. et al., 2015). TRIM26 negatively regulates interferon-β production and antiviral responses by promoting the ubiquitination and proteasomal degradation of interferon-regulatory factor 3 (IRF3) (Wang P. et al., 2015). Through mediating the ubiquitination of endonuclease VIII-like protein 1 (NEIL1), TRIM26 can increase cellular radiosensitivity in osteoblast cells (Edmonds et al., 2016). A previous study has showed that TRIM26 may serve as a novel tumor suppressor in hepatocellular carcinoma by regulating multiple metabolism-related pathways (Wang Y. et al., 2015). However, whether TRIM26 participated in HSCs activation and liver fibrosis remains to be clarified.

In the study, we investigated the expression of *TRIM26* in normal, fibrotic, and cirrhotic liver tissues, and the roles of TRIM26 in HSCs activation and liver fibrosis. In addition, we also addressed the relationship between TRIM26 and SLC7A11 in HSCs, and revealed the molecular mechanism through which TRIM26 functions in HSCs ferroptosis. Our findings propose that TRIM26-induced SLC7A11 suppression can be potentially targeted for liver fibrosis treatment.

## Materials and Methods

### Chemicals

CCl_4_ (C128126), deferoxamine (DFO) (D105648), and erastin (E126853) used in this study were purchased from Aladdin (Shanghai, China).

### Bioinformatic Analysis

The gene expression data were obtained from the Gene Expression Omnibus (GEO) dataset (Access ID: GSE25097)^1^.

### Study Subjects

The study protocol, conformed to the ethical guidelines of the 1975 Declaration of Helsinki, was approved by the Institutional Ethical Review Committee of Shanghai Ninth People’s Hospital (Shanghai, China). Written informed consents were obtained from all the participants. A total of 20 liver specimens of mild fibrosis, 20 of severe fibrosis, and 20 of cirrhosis were collected.

### Cell Culture

Human HSCs line LX-2 was obtained from the cell bank of the Shanghai Biology Institute (Chinese Academy of Sciences, Shanghai, China). The cells were cultured in RPMI-1640 medium (Hyclone) supplemented with 10% fetal bovine serum (Invitrogen) and 1% streptomycin/penicillin (Invitrogen).

### RNA Isolation and Quantitative Real-Time PCR (qRT-PCR)

Total RNA was extracted using TRIzol reagent (Invitrogen) according to the manufacturer’s instructions. The mRNA levels of indicated genes were determined by qRT-PCR using SYBR^®^ Green (Thermo Fisher Scientific) on ABI 7300 instrument (Applied Biosystems), with *GAPDH* as an internal control. All the reactions were conducted based on the following cycling parameters, 95°C for 10 min, followed by 40 cycles of 95°C for 15 s and 60°C for 45 s. Specific amplification was verified by dissociation curve analysis. Comparative C_*t*_ method was used for transcript quantification. The fold-changes of target genes, normalized by the internal control, were determined by the formula 2^–△△*CT*^. All data represent the average of triplicates. The primers are listed in Supplementary Table S1.

### Preparation of Total Cell Lysates and Western Blot Analysis

Total cell lysates were prepared with radioimmunoprecipitation assay (RIPA) buffer containing proteinase inhibitor (Beyotime), following the manufacturer’s instructions. Proteins were separated by sodium dodecyl sulfate-polyacrylamide gel electrophoresis (SDS-PAGE) and electroblotted onto nitrocellulose membranes (Millipore). After blocking with 5% skimmed milk, the membranes were incubated with primary antibodies (Supplementary Table S2) at 4°C overnight in accordance with the manufacturer’s instructions. After the unbound antibody was washed away, the membranes were further incubated with HRP-conjugated rabbit secondary antibody (Beyotime) at room temperature for 1 h. The enhanced chemiluminescence system (Millipore) was employed for signal detection.

### Construction of *TRIM26*-Overexpression Plasmids

Full-length *TRIM26* and *TRIM26* with substitute mutation of C31S were cloned into pCMV-Tag2 (Stratagene) to express Flag-tagged *TRIM26* and *TRIM26* E3 catalytic mutant (C31S) (Ran et al., 2016), respectively. The constructed plasmids were verified by double enzyme digestion and DNA sequencing.

### Construction of Adenovirus Expressing Mouse *TRIM26*

Mouse *TRIM26* was cloned into pShuttle-CMV (GenScript). The *Sal*I/*Xho*I-linearized shuttle plasmid was recombined with backbone pAdEasy-1 (GenScript) in *Escherichia coli* BJ5183 to construct pAd-TRIM26. Then, pAd-TRIM26 and pAdEasy-1 were transfected into 293 cells using Lipofectamine^TM^ 2000 reagent (Invitrogen) according to the protocol provided by the manufacturer. Recombinant adenovirus was grown and purified based on cesium chloride gradients. Viral titers were determined by plaque assay, and the virus was aliquoted for storage at −80°C.

### Lentivirus Preparation

Short hairpin RNA (shRNA) oligos targeting *TRIM26* (Supplementary Table S3) were annealed and cloned into *Age*I/*Eco*RI-digested pLKO.1 (Addgene). Full-length human *TRIM26* was cloned into pLVX-puro (Clontech). The lentivirus was produced in 293T cells along with packaging plasmids psPAX2 and pMD2.G.

### Cell Proliferation Assay

Cell proliferation was determined using the Cell Counting Kit-8 (CCK-8) Assay Kit (Jiancheng Bioengineering Institute, Nanjing, China). The LX-2 cells were cultured and treated in 96-well plates, and 10 μL of the CCK-8 reagent was added to each well for 3 h incubation in darkness. The absorbance was measured at 450 nm using a Microplate Reader. The averaged optical density (OD) of each group was used to calculate the percent of relative cell proliferation by the following formula: (OD_*treatment*_/OD_*control*_) × 100%.

### Measurement of Hydroxyproline and Glutathione (GSH) Levels

Hydroxyproline and GSH levels were measured using commercial kits (Jiancheng Bioengineering Institute) according to the manufacturer’s protocols.

### Lipid Peroxidation Assay

Lipid peroxidation was assessed using the C11-BODIPY assay kit (D3861, Thermo Fisher Scientific) according to the manufacturer’s instructions. Briefly, the cells were treated as indicated. The cultural medium was replaced with 10 mM C11-BODIPY-containing medium for 1 h. Then, the cells were harvested by trypsinization and resuspended in 1% BSA-containing PBS. The lipid ROS level was examined by flow cytometry analysis (FACSCanto^TM^ II, BD Biosciences).

Total ROS level was assessed using the Reactive Oxygen Species Assay Kit (Beyotime, S0030) and analyzed by flow cytometry.

### Measurement of MDA Content, Fe^2+^ Release Assay, and NADPH Content

MDA (malondialdehyde) content, Fe^2+^ release level, and NADPH (reduced form of nicotinamide-adenine dinucleotide phosphate) was determined using the lipid peroxidation (MDA) assay kit (Abcam, #ab118970), iron assay kit (Abcam, #ab83366), and fluorometric NADP/NADPH (Abcam, #ab65349) assay kit, respectively, following standard instructions.

### Immunoprecipitation Assay

The cell lysates were reacted with anti-TRIM26 (27013-1-AP, Invitrogen), anti-SLC7A11 (PA1-16893, Invitrogen), or control IgG (Santa Cruz Biotechnology) for 1 h at 4°C. Then, they were incubated with protein A/G-agarose for 3 h at 4°C. The precipitates were washed three times with the lysis buffer and detected by western blot analysis.

### Animal Study

Animal experiments were approved by the institutional and local animal care and use committees of the Shanghai Ninth People’s Hospital (Shanghai, China). A total of 24 C57BL/6 mice were obtained from the Sippr-BK laboratory animal Co., Ltd. (Shanghai, China). They were randomly divided into four groups: Group I, Vehicle (control); Group II, CCl_4_; Group III, CCL_4_ + Vector; Group IV, CCL_4_ + oe*TRIM26*. Liver fibrosis was induced in Groups II − IV, by intraperitoneally injecting 50% carbon tetrachloride (CCl_4_) in corn oil (0.1 mL/100 g body weight) over 8 weeks (3 times/week); control mice were injected with corn oil only. Then, recombinant adenovirus Vector or oe*TRIM26* (5 × 10^9^ pfu/mouse, 0.5 mL) was injected into the mice of Group III or IV, respectively, through the tail vein. Four weeks after the injection, all the mice were sacrificed, and blood samples were obtained for subsequent measurements of aspartate transaminase (AST), alanine transaminase (ALT), and hydroxyproline. The livers were immediately excised; half were fixed with 4% paraformaldehyde for histological examination by hematoxylin and eosin (HE), Masson’s trichrome, and Sirius red staining; the other half were frozen in liquid nitrogen for further western blot analysis. All procedures were performed in accordance with the guidelines for animal care. The qualification of Masson’s trichrome and Sirius Red staining was performed with the ImageJ software^2^ (Bethesda, MD, United States).

### Statistical Analysis

Statistical analysis was carried out using the Graphpad Prism software (version 6.0). Student’s *t*-test and analysis of variance (ANOVA) were performed to compare the data. *P*-values less than 0.05 were considered as statistically significant.

## Results

### TRIM26 Is Downregulated in Cirrhotic or Fibrotic Liver Tissues

**FIGURE 1:**
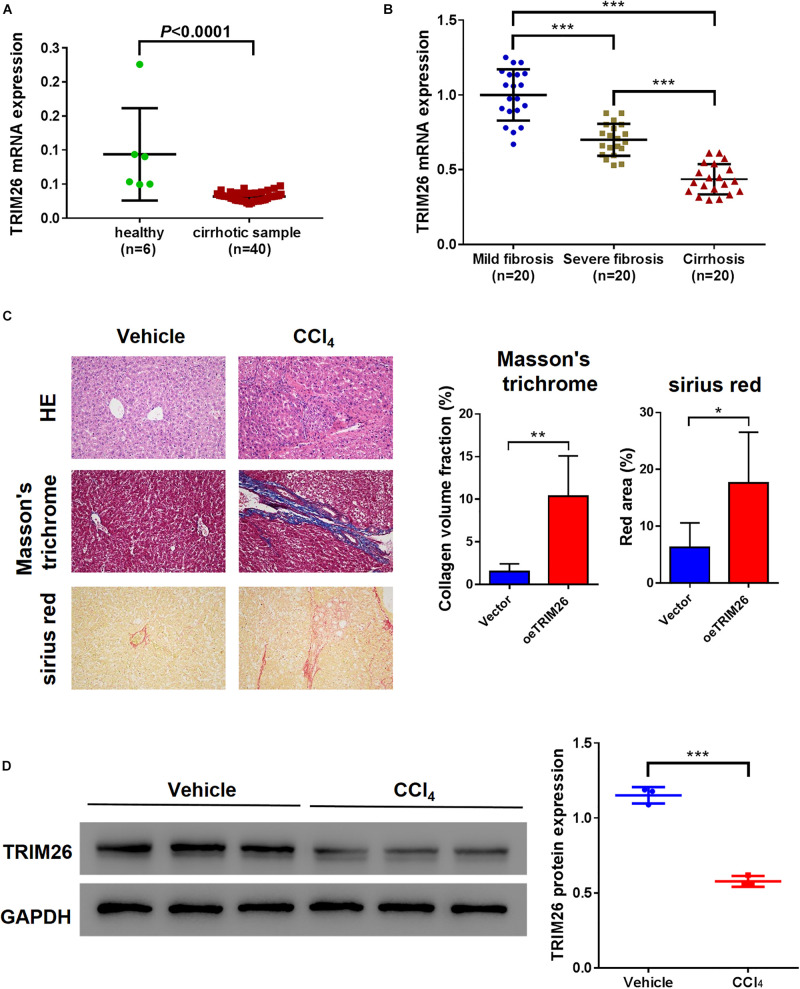
TRIM26 expression in fibrotic liver tissues. **(A)** mRNA level of *TRIM26* in normal and cirrhotic liver samples, according to GSE25097 dataset. **(B)** mRNA level of *TRIM26* in human mild-fibrotic, severe-fibrotic, and cirrhotic liver samples by qRT-PCR. **(C,D)** Mice liver fibrosis model was established by CCl_4_ induction. **(C)** histological examination (magnification at 200×) of liver tissues by hematoxylin and eosin, Masson’s trichrome, and Sirius red staining; The qualification of Masson’s trichrome and Sirius Red staining was performed with ImageJ software. **(D)** protein expression of TRIM26 by western blot analysis. *P < 0.05, **P < 0.01, ***P < 0.001.

Bioinformatic analysis based on the GSE25097 dataset revealed that the mRNA level of *TRIM26* in human cirrhotic liver tissues (*n* = 40) was significantly lower than that in normal liver samples (*n* = 6) (Figure 1A). Further, we collected liver specimens from fibrotic or cirrhotic liver patients in our hospital. Through qRT-PCR anaylsis, we observed that the mRNA level of *TRIM26* were significantly different among patient groups; the highest *TRIM26* expression was observed in those with mild liver fibrosis, followed by those with severe liver fibrosis and with liver cirrhosis (Figure 1B). Moreover, the protein expression of TRIM26 in CCl_4_-induced fibrotic liver of mice (Figure 1C) was significantly lower than that of the control (Figure 1D). Combining these findings, we demonstrate that TRIM26 is downregulated in cirrhotic or fibrotic liver.

### *TRIM26* Overexpression Promotes HSCs Ferroptosis

**FIGURE 2:**
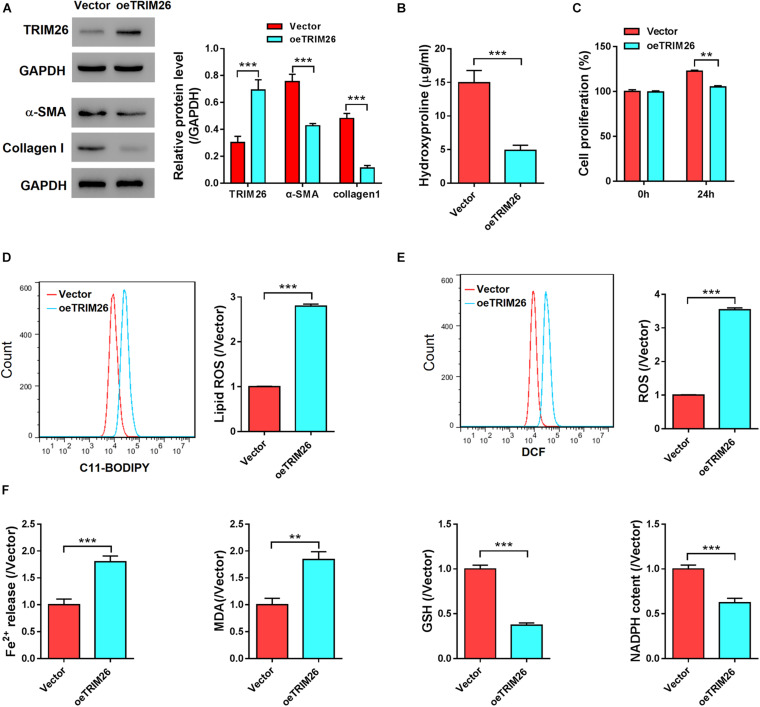
*In vitro* effect of *TRIM26* overexpression on HSCs. LX-2 cells were transfected with Vector or oe*TRIM26* plasmid. **(A)** Protein expression of TRIM26, α-SMA, and collagen I by western blot analysis. **(B)** Hydroxyproline content in culture medium by biochemical tests. **(C)** LX-2 cell proliferation by CCK-8 assay. **(D)** Lipid peroxidation level by using the C11-BODIPY assay kit. **(E)** Total ROS level by using Reactive Oxygen Species Assay Kit. **(F)** Fe^2+^ release, MDA content, GSH level, and NADPH level by biochemical tests. **P < 0.01, ***P < 0.001.

To investigate the effect of TRIM26 on HSCs, oe*TRIM26* plasmid was transduced into LX-2 cells, which upregulated the protein expression of TRIM26 (Figure 2A and Supplementary Figure S1A). Western blot analysis was then performed to detected the expression of α-smooth muscle actin (α-SMA), a marker of HSCs activation (Zhao et al., 2020), and collagen I, a marker of fibrosis (Schuppan, 2015). The results revealed that *TRIM26* overexpression led to the downregulation of α-SMA and collagen I at the protein level (Figure 2A). Through biochemical tests, we detected that the cellular levels of hydroxyproline, a marker of collagen production (Schuppan, 2015), in *TRIM26*-overexpressed LX-2 cells were substantially lower than those of the controls (Figure 2B). Furthermore, the overexpressed *TRIM26* substantially inhibited LX-2 cell proliferation by approximately 15% (Figure 2C), while increased the lipid ROS level by nearly threefold (Figure 2D) and the total ROS level (Figure 2E) by more than threefold. Additionally, we also examined Fe^2+^ release, the cellular level of MDA, GSH, and NADPH to verify the occurrence of ferroptosis. We found that Fe^2+^ release and MDA content was elevated, while GSH and NAPDH was substantially downregulated in *TRIM26*-overexpressed LX-2 (Figure 2F).

**FIGURE 3:**
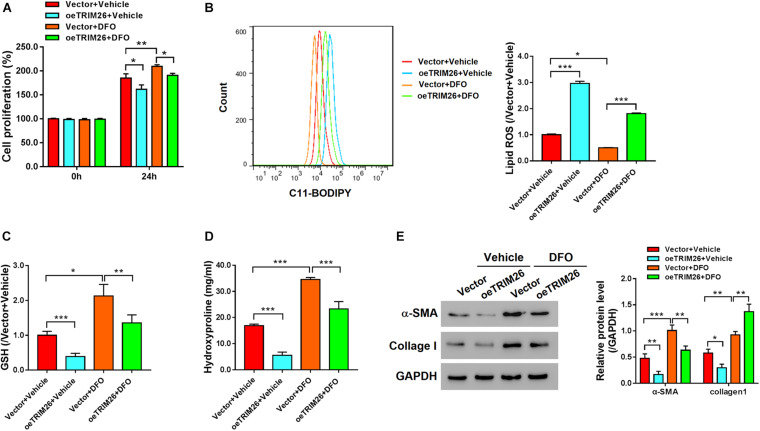
Effect of *TRIM26* overexpression on deferoxamine (DFO)-inhibited fibrosis. LX-2 cells were treated with 100 μM DFO or Vehicle after 24 h of Vector/oe*TRIM26* transfection. **(A)** Cell proliferation. **(B)** Lipid peroxidation level. **(C)** Glutathione content. **(D)** Hydroxyproline content. **(E)** Protein expressions of α-SMA and collagen I. *P < 0.05; **P < 0.01; ***P < 0.001.

We further treated the LX-2 cells with iron inhibitor DFO, to examine the function of TRIM26 on HSCs ferroptosis. Compared with the untreated LX-2 cells, we observed that the proliferation of LX-2 cells treated with 100 μM DFO was substantially accelerated (Figure 3A), while their lipid ROS levels (Figure 3B) were substantially lower and cellular levels of GSH were significantly increased (Figure 3C). Moreover, DFO treatment also increased the levels of hydroxyproline (Figure 3D), α-SMA, and collagen I (Figure 3E) in the LX-2 cells. However, *TRIM26* overexpression could contract the inhibitory effect of DFO on HSCs ferroptosis. At one hand, *TRIM26* overexpression substantially reversed the proliferation rate (Figure 3A) and lipid ROS level (Figure 3B) of the LX-2 cells treated with DFO; at the other hand, under DFO treatment, *TRIM26*-overexpressed LX-2 cells exhibited lower levels of hydroxyproline (Figure 3D), α-SMA, and collagen I (Figure 3E) in comparison with the controls. Such findings demonstrate the function of TRIM26 in facilitating HSCs ferroptosis.

### TRIM26 Facilitates SLC7A11 Ubiquitination

**FIGURE 4:**
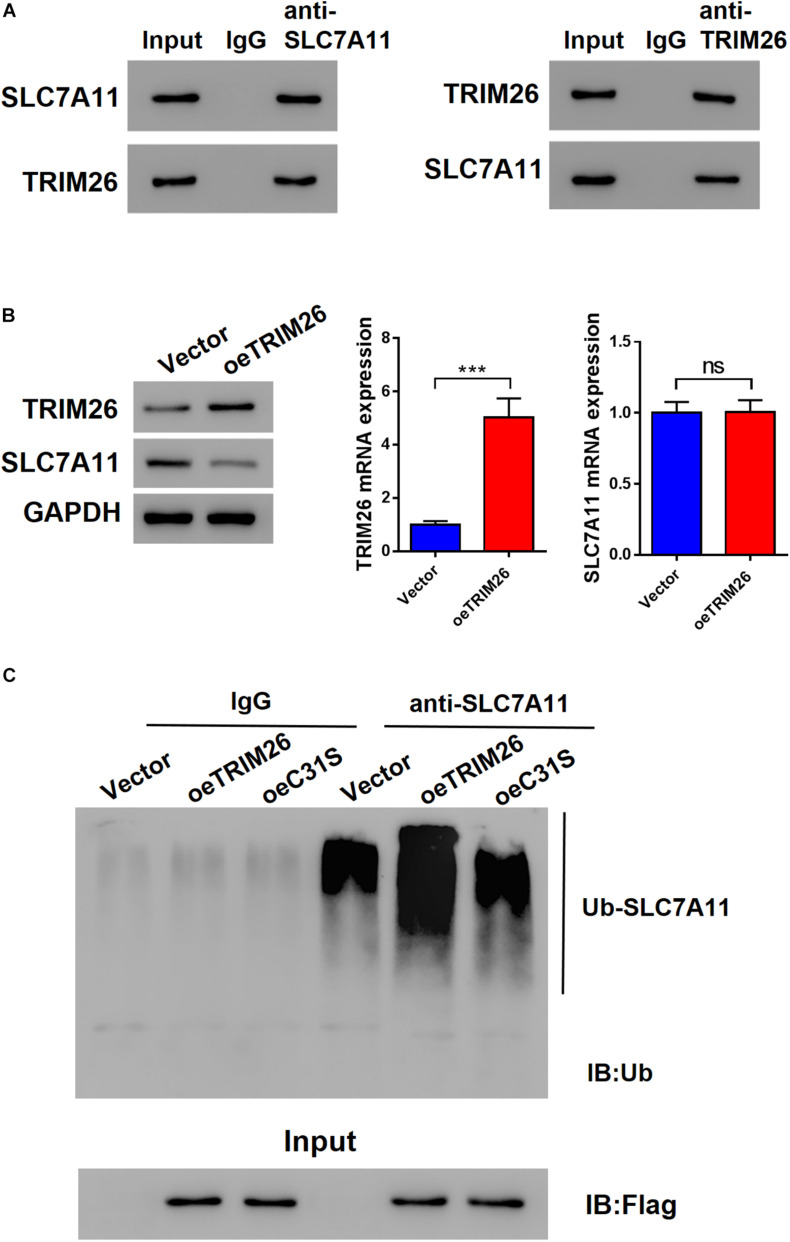
Interactions between TRIM26 and SLC7A11. **(A)** Co-immunoprecipitation of TRIM26 and SLC7A11 in LX-2 cells. **(B)** Protein and mRNA expressions of TRIM26 and SLC7A11 in Vector- or oe*TRIM26* plasmid-transfected LX-2 cells. **(C)** SLC7A11 ubiquitination in Vector-, oe*TRIM26* plasmid, or oe*C31S* plasmid-transfected LX-2 cells. ***P < 0.001. ns: not significant.

To identify the TRIM26-interacting proteins, we performed mass spectrometry-based proteomics analysis on the co-immunoprecipitation (co-IP) sample, in which SLC7A11 ranked high among the candidate interacting proteins of TRIM26 (Supplementary Table S4 and Supplementary Figure S2). Further, co-IP experiments with the LX-2 cell lysates confirmed that TRIM26 and SLC7A11 could physically interact with each other (Figure 4A). When TRIM26 was overexpressed in LX-2 cells, the protein expression of SLC7A11 was downregulated, while its mRNA level remained unchanged (Figure 4B). Therefore, we speculated that TRIM26 could regulate SLC7A11 merely at the protein level. Through further investigation, we identified that *TRIM26* overexpression in LX-2 cells could enhance the ubiquitination of SLC7A11, while no such effect was observed in the cells overexpressed with E3 catalytic mutant TRIM26 (C31S) (Figure 4C). These findings indicate that TRIM26 can facilitate SLC7A11 ubiquitination, which was dependent on the E3 ligase activity of TRIM26.

### TRIM26 Induces HSCs Ferroptosis Through Regulating SLC7A11

**FIGURE 5:**
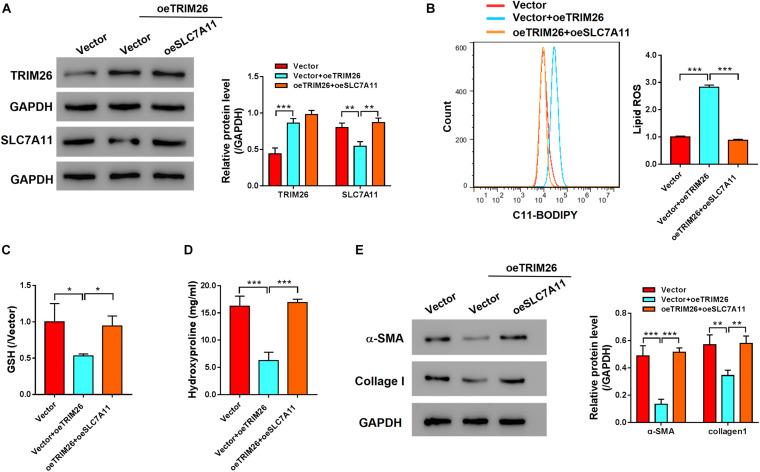
SLC7A11-dependent mechanism of LX-2 ferroptosis. LX-2 cells were co-transfected with oe*TRIM26* and oe*SLC7A11* plasmids. **(A)** Protein expressions of TRIM26 and SLC7A11. **(B)** Lipid peroxidation level. **(C)** Glutathione content. **(D)** Hydroxyproline content. **(E)** Protein expressions of α-SMA and collagen I. *P < 0.05; **p < 0.01; ***P < 0.001.

To further investigate the role of SLC7A11 in TRIM26-induced ferroptosis, oeSLC7A11 and oeTRIM26 plasmids were co-transfected into LX-2 cells. We identified that the protein expression of SLC7A11 was downregulated in TRIM26-overexpressed LX-2 cells, while it was reversed by SLC7A11 overexpression (Figure 5A and Supplementary Figure S2). In comparison with the LX-2 cells with TRIM26 overexpression alone, oeSLC7A11 and oeTRIM26 co-transfection significantly restored lipid ROS back to the normal level of the control (Figure 5B). Meanwhile, SLC7A11 overexpression also significantly counteracted the inhibitory effect of TRIM26 overexpression on ferroptosis indicator molecules in LX-2 cells; the cellular levels of GSH (Figure 5C) and hydroxyproline (Figure 5D) in the LX-2 cells with oeSLC7A11 and oeTRIM26 co-transfection were significantly higher than those with oeTRIM26 transfection only; similarly, the protein expressions of α-SMA and collagen I were upregulated (Figure 5E). Therefore, we speculate that the ferroptosis-promotive effect of TRIM26 is through regulating SLC7A11.

### TRIM26 Overexpression Attenuates CCl_4_-Induced Liver Fibrosis

**FIGURE 6:**
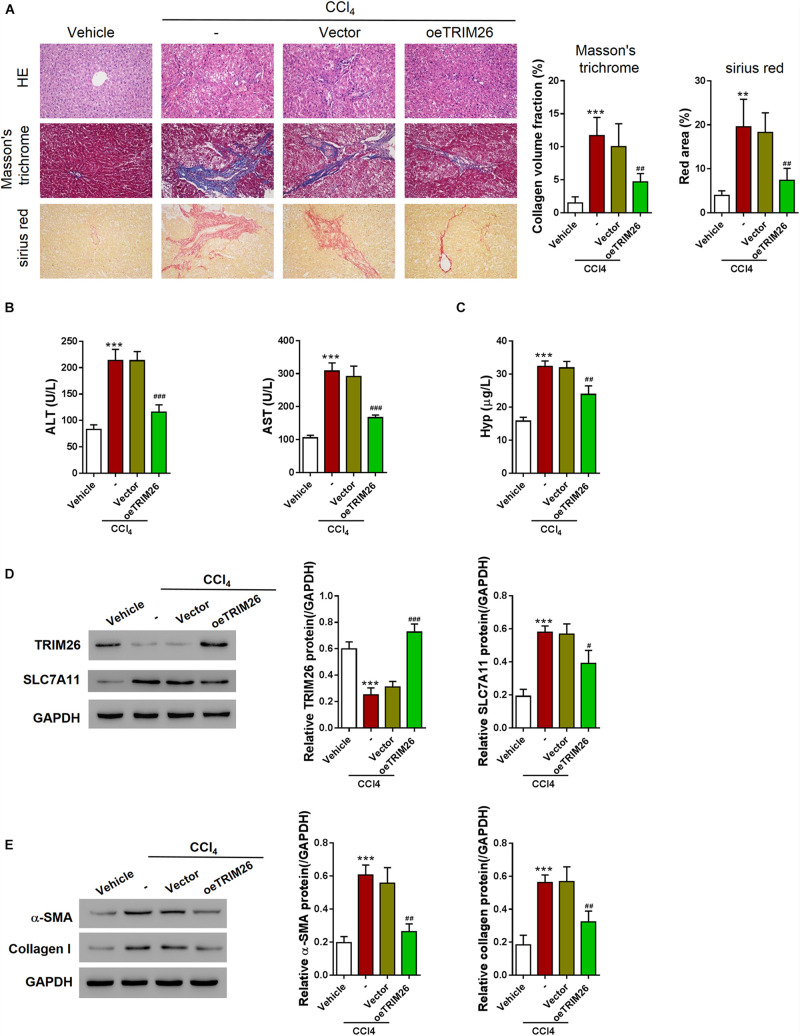
*In vivo* effect of *TRIM26* overexpression on CCl_4_-induced liver fibrosis. Mice liver fibrosis model was established by CCl_4_ induction. Recombinant adenovirus Vector or oe*TRIM26* was injected into the mice through tail vein. **(A)** Histological examination (magnification at 200×) of liver tissues by hematoxylin and eosin, Masson’s trichrome, and Sirius red staining. **(B)** The serum levels of alanine transaminase (ALT) and aspartate transaminase (AST). **(C)** The serum levels of hydroxyproline (Hyp). **(D)** Proteins expressions of TRIM26 and SLC7A11 in liver tissues. **(E)** Protein expressions of α-SMA and collagen I in liver tissues. **p < 0.01; ***P < 0.001 vs. Vehicle; ^#^*P* < 0.05, ^##^*P* < 0.01, and ^###^*P* < 0.001 vs. CCl_4_ + Vector.

After ferroptosis-promotive effect of TRIM26 was revealed *in vitro*, we next designated to examine its protective effect on liver fibrosis *in vivo*. Through intraperitoneally injecting CCl_4_ into mice, we successfully established a liver fibrosis animal model. Based on morphological examinations with H&E, Masson’s trichrome, and Sirius red staining, we observed that CCl_4_ injection induced extensive fibrotic deposition in liver tissues of the mice. However, the mice with oeTRIM26 adenovirus administration exhibited mild liver fibrosis (Figure 6A). At the meantime, CCl_4_ considerably increased the serum levels of ALT, AST, and hydroxyproline (Figures 6B,C); while in contrast, the overexpression of TRIM26 effectively counteracted CCl_4_’s effect by reducing ALT, AST (Figure 6B), and hydroxyproline (Figure 6C). Following a similar trend, CCl_4_ injection downregulated the protein level of TRIM26 (Figure 6D), while upregulated SLC7A11 (Figure 6D), α-SMA, and collagen I (Figure 6E); in contrast, TRIM26 overexpression restored the expressions of these indicator proteins, i.e., a higher protein level of TRIM26 (Figure 6D) and lower protein levels of SLC7A11 (Figure 6D), α-SMA, and collagen I (Figure 6E). Overall, these observations reveal that TRIM26 overexpression can mitigate liver fibrosis triggered by CCl_4_ in mice.

## Discussion

The connection between TRIM26 and hepatocellular carcinoma has been explored previously (Wang Y. et al., 2015). For this study, we evaluated the expression pattern of *TRIM26* based on the GEO dataset, and detected its downregulation in cirrhotic liver (Figure 1). Through examining the gene expression by qRT-PCR, we further confirmed the downregulation pattern of *TRIM26* in liver tissue specimens from patients with fibrotic or cirrhotic liver (Figure 1). Moreover, the translational level of *TRIM26* is reversely proportional to the severity of liver fibrosis in liver fibrosis animal model (Figure 1), indicating the correlation between TRIM26 expression and liver fibrotic progression.

Several studies have reported that E3 ubiquitin ligases, such as Smurf2, HectD3, and NEDD4, could inhibit collagen deposition (Cai et al., 2018; Huang et al., 2020; Rangrez et al., 2020). Consistent with these findings, we found that LX-2 cells with *TRIM26* overexpression were associated with a lower expression of hydroxyproline, α-SMA, and collagen I (Figure 2). Hydroxyproline is converted from proline through post-translational hydroxylation during collagen biosynthesis (Wu et al., 2011); α-SMA upregulation reflects fibroblast activation and associates with liver fibrogenesis (Zhao et al., 2020); collagen I is continuously secreted by activated HSCs to accumulate, forming fibrotic scarring (Schuppan, 2015). Collectively, reduction of these critical indicators of liver fibrosis reveals that *TRIM26* overexpression inhibits HSCs activation and the fibrogenic process, which facilitated us to explore its underlying mechanism. The stimulated lipid peroxidation and suppressed cell proliferation are common features of ferroptosis (Imai et al., 2017; Li et al., 2020), and were found to be induced by *TRIM26* overexpression in LX-2 cells (Figure 2). Meanwhile, the increased level of lipid ROS was in line with the downregulated expression of GSH (Figure 2), both of which suggest that TRIM26 might participate in HSCs ferroptosis.

The potent iron chelator DFO used for treating iron overload has been recognized as an effective ferroptosis inhibitor (Yao et al., 2019). Here in the present study, *TRIM26* overexpression counteracted DFO’s inhibitory effect on ferroptosis (Figure 3), which further confirms the role of TRIM26 in facilitating HSCs ferroptosis. On the other hand, erastin can induce ferroptotic cell death through facilitating ROS accumulation (Sun et al., 2020; Zhao et al., 2020). We triggered LX-2 cell ferroptosis by implying erastin, while found that *TRIM26* knockdown could counteract erastin’s promotive effect on ferroptosis (Supplementary Figure S4). However, the findings from our study cannot exclude the possibility that TRIM26 may regulate other forms of programmed cell death as well (Friedman and Bansal, 2006; Thoen et al., 2011). Further studies are warranted to examine additional mechanisms underlying TRIM26’s inhibitory effect on HSCs activation, for example, through inducing apoptosis and autophagy.

The Xc^–^ antiporter system mediates intracellular glutamate and extracellular cystine exchange for anti-oxidant reactions (Lewerenz et al., 2013); inhibiting system Xc^–^ can induce intracellular GSH depletion and subsequent iron-dependent lipid peroxidation (i.e., ferroptosis) (Dixon et al., 2014). The expression of SLC7A11, a light chain subunit of system Xc^–^, has been positively correlated with the antiporter activity (Lewerenz et al., 2013; Wang et al., 2020). Previously, the small molecule erastin was used as a selective inhibitor of SLC7A11 for disrupting glutamate-cystine exchange and triggering ferroptosis (Dixon et al., 2014; Wang et al., 2020). Considering the vital role of SLC7A11 in system Xc^–^ and ferroptosis, we examined its relationship with TRIM26, and revealed that TRIM26 not only can physically interact with SLC7A11, but also mediates the ubiquitination and degradation of SLC7A11 in HSCs (Figure 4). Previously, deubiquitylase OTUB1 has been recognized to stabilize SLC7A11 and suppress the activation of ferroptosis (Liu et al., 2019), whereas potential ubiquitin ligases that mediate SLC7A11 ubiquitination have not been identified (Koppula et al., 2020). The findings from this study provide a candidate E3 ubiquitin ligase, TRIM26, for post-translational regulation of SLC7A11 and system Xc^–^. However, the specific molecular mechanism and potential co-factors of TRIM26-mediated SLC7A11 ubiquitination and proteasomal degradation remains to be further discovered.

Furthermore, our study, for the first time, reports the anti-fibrotic role of TRIM26 through regulating SLC7A11, thus enhancing ferroptosis of the activated HSCs. To be noted, the toxicological mechanism of CCl_4_-induced liver fibrosis has been recently discovered (Dong et al., 2016), based on which multiple researches established experimental liver fibrosis in animals such as mice and rats (Fujii et al., 2010; Scholten et al., 2015). Accordantly, by CCl_4_-induction, we successfully established a liver fibrotic mice model, and relying on this experimental model, we were able to demonstrate the function of TRIM26 *in vivo*. Overall, this study reveals the potent therapeutic effectiveness of TRIM26 on liver fibrosis, while further study might be necessary to investigate the long-term function of *TRIM26* overexpression and the potential adverse effects it may introduce.

## Conclusion

**FIGURE 7:**
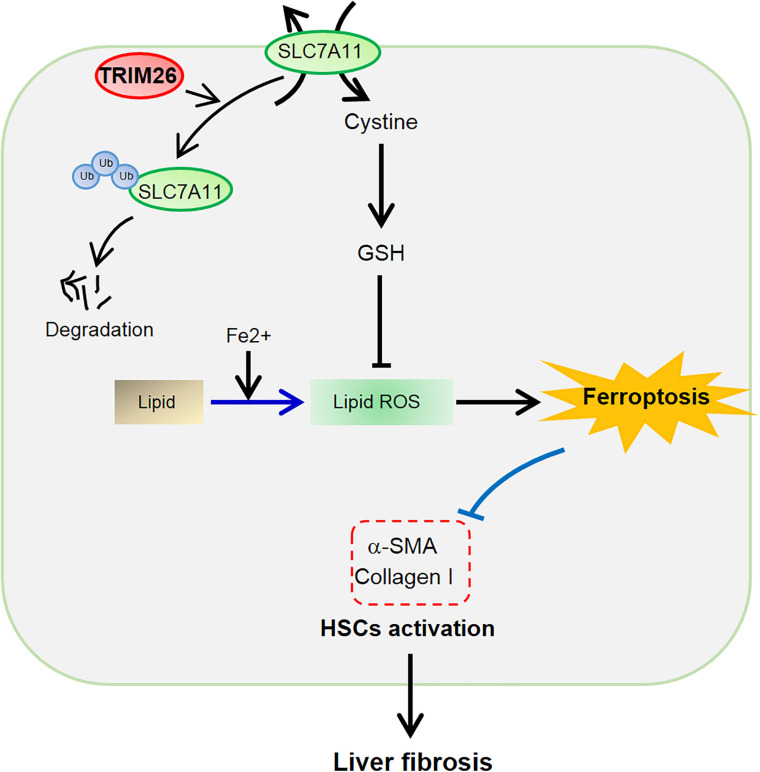
Schematic of the TRIM26-involved regulation of HSCs ferroptosis and activation during liver fibrosis.

In conclusion, TRIM26 is critical for HSCs ferroptosis to attenuate liver fibrosis. It is downregulated in fibrotic liver tissues, while the overexpression of *TRIM26* induces lipid ROS accumulation, leading to the ferroptosis of activated HSCs. The mechanistic study reveals that TRIM26 promotes HSCs ferroptosis through mediating SLC7A11 ubiquitination and degradation (Figure 7). Furthermore, we also demonstrate that *TRIM26* overexpression in mice can suppress SLC7A11 and effectively mitigate CCl_4_-induced liver fibrosis. Although the functions of TRIM26 in other forms of HSCs programmed cell death need to be further investigated, the findings from the current study provide novel insights on an SLC7A11-targeted therapeutic approach for hepatic fibrosis.

## Data Availability Statement

The original contributions presented in the study are included in the article/Supplementary Material, further inquiries can be directed to the corresponding author/s.

## Ethics Statement

The studies involving human participants were reviewed and approved by the Institutional Ethical Review Committee of Shanghai Ninth People’s Hospital (Shanghai, China). The patients/participants provided their written informed consent to participate in this study. The animal study was reviewed and approved by the Animal Care and Use Committees of Shanghai Ninth People’s Hospital (Shanghai, China).

## Author Contributions

YZ and TN designed the experiments. YZ, CZ, and MH performed the experiments. YZ, JL, and XF performed the statistical analysis. YZ wrote the manuscript. TN supervised the study. All authors have read and approved the final version of the manuscript.

## Conflict of Interest

The authors declare that the research was conducted in the absence of any commercial or financial relationships that could be construed as a potential conflict of interest.

## Footnotes

1 http://www.ncbi.nlm.nih.gov

2 http://rsb.info.nih.gov/ij/
