# Supplementary material for: TRIM26 Induces Ferroptosis to Inhibit Hepatic Stellate Cell Activation and Mitigate Liver Fibrosis Through Mediating SLC7A11 Ubiquitination
Source: Front Cell Dev Biol. 2021 Mar 25;9:644901. doi: 10.3389/fcell.2021.644901 (PMC8044755; doi:10.3389/fcell.2021.644901)
Supplement: Supplementary file 1 [file Data_Sheet_1.DOCX]

**
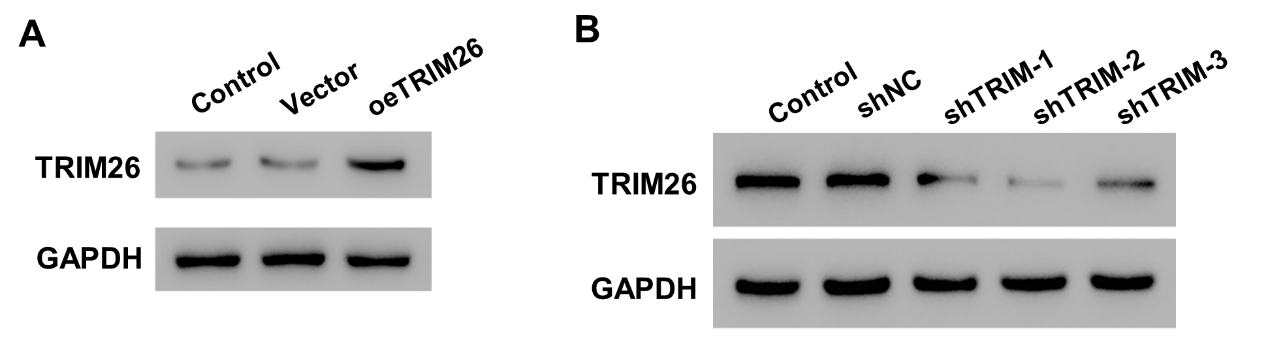
**

**Figure S1.** Manipulation of TRIM26 in LX-2 cells. (A) TRIM26 protein expression in wild-type LX-2 cells or those transfected with Vector or oe*TRIM26* plasmid. (B) TRIM26 protein expression in wild-type LX-2 cells or those transduced with shNC or shTRIM-1/2/3.


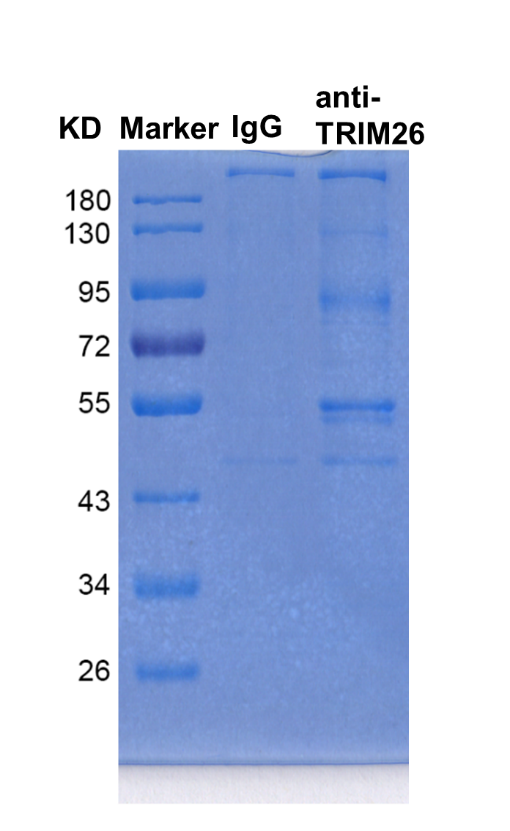


**Figure S2.** Protein bands for the immunoprecipitated complexes formed in LX-2 cell lysates.


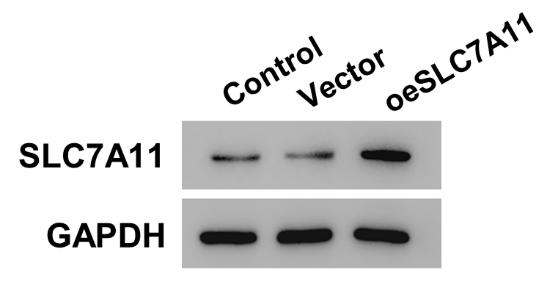


**Figure S3.** Overexpression of SLC7A11 in LX-2 cells. SLC7A11 protein expression in wild-type LX-2 cells or those transfected with Vector or oe*TRIM26* plasmid.

**Figure S4.** Effect of *TRIM26* knockdown on erastin-induced ferroptosis. LX-2 cells were treated with 10 μM erastin or Vehicle after 24 hours of shNC/shTRIM-2 transduction. (A) Cell proliferation. (B) Lipid peroxidation level. (C) Glutathione content. (D) Protein expressions of GPX4 and SLC7A11. * P < 0.05; ** P < 0.01; *** P < 0.001.
