# Supplementary material for: TRIM26 Induces Ferroptosis to Inhibit Hepatic Stellate Cell Activation and Mitigate Liver Fibrosis Through Mediating SLC7A11 Ubiquitination
Source: Front Cell Dev Biol. 2021 Mar 25;9:644901. doi: 10.3389/fcell.2021.644901 (PMC8044755; doi:10.3389/fcell.2021.644901)
Supplement: Supplementary file 2 [file Data_Sheet_2.DOCX]

**Materials and methods**

**Immunoprecipitation (IP) and liquid chromatography/mass spectrometry (LC/MS) analysis**

The LX-2 cells were extracted using the RIPA buffer. Following incubation with IgG and protein A/G beads (Santa Cruz Biotechnology) at 4°C for 2 h, the cell extracts were incubated with anti-TRIM26 (27013-1-AP, Invitrogen) or control IgG (Santa Cruz Biotechnology) supplemented with protein A/G-agarose overnight at 4°C. The immunoprecipitated protein complexes were resolved on SDS-PAGE and stained with Coomassie Brilliant Blue. Differential bands were excised, digested with trypsin, and analyzed by LC/MS.

**Table S1.** Primer sequences for real-time PCR.

| **Gene** | **Forward primer** | **Reverse primer** |
| --- | --- | --- |
| TRIM26 | 5' GAGGAGGAAGTTCTGGAAAG 3' | 5' TCTGCGTTGGTGAAAGTC 3' |
| SLC7A11 | 5' CTAACTAACTGGTCCTCAACTC 3' | 5' CCTAAGAAACAACGCAATCC 3' |
| GAPDH | 5' AATCCCATCACCATCTTC 3' | 5' AGGCTGTTGTCATACTTC 3' |

**Table S2.** Antibody used in the study.

| **Primary antibody** | **Company** | **Catalog No.** |
| --- | --- | --- |
| TRIM26 | Abcam | Ab89290 |
| α-SMA | Abcam | Ab32575 |
| Collagen I | Abcam | Ab90395 |
| SLC7A11 | Invitrogen | PA5-18599 |
| GPX4 | Abcam | Ab125066 |
| GAPDH | Cell Signaling Technology | #5174 |

**Table S3.** Target sequences of human *TRIM26* shRNAs.

| **shRNA** | **Target sequence** |
| --- | --- |
| shTRIM-1 | 5’ GGCAAAGGGAGAAGCTGAT 3’ |
| shTRIM-2 | 5’ GCCTGTACAAGAGTGCCTA 3’ |
| shTRIM-3 | 5’ GCAGTTTGACTGTGAGCCT 3’ |

**Table S4.** Identification of TRIM26-interacting proteins by IP and LC/MS anslysis.

| Accession | Description | Score | Coverage | # Proteins | # Unique Peptides | # Peptides | # PSMs | # AAs | MW [kDa] | calc. pI |
| --- | --- | --- | --- | --- | --- | --- | --- | --- | --- | --- |
| Q9UPY5 | Solute Carrier Family 7 Member 11 OS=Homo sapiens GN=SLC7A11 PE=1 SV=6 - [SLC7A11_HUMAN] | 210.92 | 25.78 | 1 | 15 | 21 | 28 | 644 | 55.4 | 9.51 |
| P67936 | Tropomyosin alpha-4 chain OS=Homo sapiens GN=TPM4 PE=1 SV=3 - [TPM4_HUMAN] | 306.17 | 43.95 | 2 | 8 | 14 | 20 | 248 | 28.5 | 4.69 |
| P23396 | 40S ribosomal protein S3 OS=Homo sapiens GN=RPS3 PE=1 SV=2 - [RS3_HUMAN] | 257.76 | 50.62 | 1 | 14 | 14 | 24 | 243 | 26.7 | 9.66 |
| P18124 | 60S ribosomal protein L7 OS=Homo sapiens GN=RPL7 PE=1 SV=1 - [RL7_HUMAN] | 190.77 | 39.92 | 2 | 13 | 13 | 18 | 248 | 29.2 | 10.65 |
| P25786 | Proteasome subunit alpha type-1 OS=Homo sapiens GN=PSMA1 PE=1 SV=1 - [PSA1_HUMAN] | 192.88 | 39.54 | 1 | 12 | 12 | 15 | 263 | 29.5 | 6.61 |
| P09525 | Annexin A4 OS=Homo sapiens GN=ANXA4 PE=1 SV=4 - [ANXA4_HUMAN] | 269.42 | 32.60 | 1 | 10 | 11 | 16 | 319 | 35.9 | 6.13 |
| P62258 | 14-3-3 protein epsilon OS=Homo sapiens GN=YWHAE PE=1 SV=1 - [1433E_HUMAN] | 200.00 | 30.98 | 1 | 10 | 11 | 16 | 255 | 29.2 | 4.74 |
| P06753 | Tropomyosin alpha-3 chain OS=Homo sapiens GN=TPM3 PE=1 SV=2 - [TPM3_HUMAN] | 189.68 | 31.58 | 1 | 3 | 10 | 17 | 285 | 32.9 | 4.72 |
| P02768 | Serum albumin OS=Homo sapiens GN=ALB PE=1 SV=2 - [ALBU_HUMAN] | 135.38 | 13.96 | 1 | 10 | 10 | 10 | 609 | 69.3 | 6.28 |
| P15559 | NAD(P)H dehydrogenase [quinone] 1 OS=Homo sapiens GN=NQO1 PE=1 SV=1 - [NQO1_HUMAN] | 316.34 | 25.55 | 1 | 9 | 9 | 28 | 274 | 30.8 | 8.88 |
| P08758 | Annexin A5 OS=Homo sapiens GN=ANXA5 PE=1 SV=2 - [ANXA5_HUMAN] | 201.18 | 23.44 | 1 | 8 | 9 | 12 | 320 | 35.9 | 5.05 |
| P62424 | 60S ribosomal protein L7a OS=Homo sapiens GN=RPL7A PE=1 SV=2 - [RL7A_HUMAN] | 126.45 | 23.31 | 1 | 9 | 9 | 10 | 266 | 30.0 | 10.61 |
| P02533 | Keratin, type I cytoskeletal 14 OS=Homo sapiens GN=KRT14 PE=1 SV=4 - [K1C14_HUMAN] | 277.82 | 15.04 | 14 | 2 | 8 | 12 | 472 | 51.5 | 5.16 |
| P17931 | Galectin-3 OS=Homo sapiens GN=LGALS3 PE=1 SV=5 - [LEG3_HUMAN] | 160.03 | 29.20 | 1 | 8 | 8 | 22 | 250 | 26.1 | 8.56 |
| P08727 | Keratin, type I cytoskeletal 19 OS=Homo sapiens GN=KRT19 PE=1 SV=4 - [K1C19_HUMAN] | 135.65 | 16.00 | 12 | 2 | 8 | 8 | 400 | 44.1 | 5.14 |
| P05783 | Keratin, type I cytoskeletal 18 OS=Homo sapiens GN=KRT18 PE=1 SV=2 - [K1C18_HUMAN] | 121.39 | 12.09 | 8 | 6 | 8 | 8 | 430 | 48.0 | 5.45 |
| P04259 | Keratin, type II cytoskeletal 6B OS=Homo sapiens GN=KRT6B PE=1 SV=5 - [K2C6B_HUMAN] | 106.90 | 9.40 | 8 | 1 | 8 | 10 | 564 | 60.0 | 8.00 |
| O43399 | Tumor protein D54 OS=Homo sapiens GN=TPD52L2 PE=1 SV=2 - [TPD54_HUMAN] | 102.43 | 29.13 | 1 | 8 | 8 | 9 | 206 | 22.2 | 5.36 |
| P08779 | Keratin, type I cytoskeletal 16 OS=Homo sapiens GN=KRT16 PE=1 SV=4 - [K1C16_HUMAN] | 223.25 | 11.63 | 13 | 1 | 7 | 11 | 473 | 51.2 | 5.05 |
| P09497 | Clathrin light chain B OS=Homo sapiens GN=CLTB PE=1 SV=1 - [CLCB_HUMAN] | 113.70 | 24.89 | 1 | 7 | 7 | 7 | 229 | 25.2 | 4.64 |
| P60709 | Actin, cytoplasmic 1 OS=Homo sapiens GN=ACTB PE=1 SV=1 - [ACTB_HUMAN] | 100.12 | 16.53 | 11 | 7 | 7 | 8 | 375 | 41.7 | 5.48 |
| P27105 | Erythrocyte band 7 integral membrane protein OS=Homo sapiens GN=STOM PE=1 SV=3 - [STOM_HUMAN] | 97.16 | 27.08 | 2 | 7 | 7 | 9 | 288 | 31.7 | 7.88 |
| P40926 | Malate dehydrogenase, mitochondrial OS=Homo sapiens GN=MDH2 PE=1 SV=3 - [MDHM_HUMAN] | 95.97 | 21.30 | 1 | 7 | 7 | 11 | 338 | 35.5 | 8.68 |
| P12004 | Proliferating cell nuclear antigen OS=Homo sapiens GN=PCNA PE=1 SV=1 - [PCNA_HUMAN] | 40.61 | 22.61 | 1 | 7 | 7 | 8 | 261 | 28.8 | 4.69 |
| Q9BRP8 | Partner of Y14 and mago OS=Homo sapiens GN=WIBG PE=1 SV=1 - [WIBG_HUMAN] | 164.58 | 25.49 | 1 | 6 | 6 | 9 | 204 | 22.6 | 9.45 |
| P00338 | L-lactate dehydrogenase A chain OS=Homo sapiens GN=LDHA PE=1 SV=2 - [LDHA_HUMAN] | 160.31 | 16.27 | 1 | 6 | 6 | 10 | 332 | 36.7 | 8.27 |
| P09493 | Tropomyosin alpha-1 chain OS=Homo sapiens GN=TPM1 PE=1 SV=2 - [TPM1_HUMAN] | 133.25 | 19.01 | 1 | 1 | 6 | 10 | 284 | 32.7 | 4.74 |
| P35232 | Prohibitin OS=Homo sapiens GN=PHB PE=1 SV=1 - [PHB_HUMAN] | 123.51 | 22.06 | 1 | 6 | 6 | 6 | 272 | 29.8 | 5.76 |
| P61247 | 40S ribosomal protein S3a OS=Homo sapiens GN=RPS3A PE=1 SV=2 - [RS3A_HUMAN] | 114.93 | 24.62 | 1 | 6 | 6 | 7 | 264 | 29.9 | 9.73 |
| P62873 | Guanine nucleotide-binding protein G(I)/G(S)/G(T) subunit beta-1 OS=Homo sapiens GN=GNB1 PE=1 SV=3 - [GBB1_HUMAN] | 106.48 | 17.65 | 4 | 6 | 6 | 7 | 340 | 37.4 | 6.00 |
| P15880 | 40S ribosomal protein S2 OS=Homo sapiens GN=RPS2 PE=1 SV=2 - [RS2_HUMAN] | 101.00 | 16.38 | 1 | 6 | 6 | 6 | 293 | 31.3 | 10.24 |
| P14314 | Glucosidase 2 subunit beta OS=Homo sapiens GN=PRKCSH PE=1 SV=2 - [GLU2B_HUMAN] | 68.35 | 9.28 | 1 | 6 | 6 | 7 | 528 | 59.4 | 4.41 |
| Q15056 | Eukaryotic translation initiation factor 4H OS=Homo sapiens GN=EIF4H PE=1 SV=5 - [IF4H_HUMAN] | 63.69 | 21.37 | 2 | 6 | 6 | 6 | 248 | 27.4 | 7.23 |
| Q06830 | Peroxiredoxin-1 OS=Homo sapiens GN=PRDX1 PE=1 SV=1 - [PRDX1_HUMAN] | 40.09 | 29.65 | 1 | 5 | 6 | 7 | 199 | 22.1 | 8.13 |
| Q00688 | Peptidyl-prolyl cis-trans isomerase FKBP3 OS=Homo sapiens GN=FKBP3 PE=1 SV=1 - [FKBP3_HUMAN] | 130.54 | 18.30 | 1 | 5 | 5 | 8 | 224 | 25.2 | 9.28 |
| P62753 | 40S ribosomal protein S6 OS=Homo sapiens GN=RPS6 PE=1 SV=1 - [RS6_HUMAN] | 108.68 | 17.67 | 1 | 5 | 5 | 6 | 249 | 28.7 | 10.84 |
| P07355 | Annexin A2 OS=Homo sapiens GN=ANXA2 PE=1 SV=2 - [ANXA2_HUMAN] | 103.91 | 13.86 | 2 | 5 | 5 | 5 | 339 | 38.6 | 7.75 |
| Q16698 | 2,4-dienoyl-CoA reductase, mitochondrial OS=Homo sapiens GN=DECR1 PE=1 SV=1 - [DECR_HUMAN] | 80.93 | 15.52 | 1 | 5 | 5 | 7 | 335 | 36.0 | 9.28 |
| P68104 | Elongation factor 1-alpha 1 OS=Homo sapiens GN=EEF1A1 PE=1 SV=1 - [EF1A1_HUMAN] | 79.91 | 9.96 | 3 | 5 | 5 | 6 | 462 | 50.1 | 9.01 |
| Q06323 | Proteasome activator complex subunit 1 OS=Homo sapiens GN=PSME1 PE=1 SV=1 - [PSME1_HUMAN] | 71.97 | 23.29 | 1 | 5 | 5 | 6 | 249 | 28.7 | 6.02 |
| Q96CT7 | Coiled-coil domain-containing protein 124 OS=Homo sapiens GN=CCDC124 PE=1 SV=1 - [CC124_HUMAN] | 70.10 | 16.14 | 1 | 5 | 5 | 6 | 223 | 25.8 | 9.54 |
| O95721 | Synaptosomal-associated protein 29 OS=Homo sapiens GN=SNAP29 PE=1 SV=1 - [SNP29_HUMAN] | 68.78 | 24.42 | 1 | 5 | 5 | 7 | 258 | 29.0 | 5.81 |
| P63244 | Guanine nucleotide-binding protein subunit beta-2-like 1 OS=Homo sapiens GN=GNB2L1 PE=1 SV=3 - [GBLP_HUMAN] | 68.27 | 12.93 | 1 | 5 | 5 | 5 | 317 | 35.1 | 7.69 |
| Q13011 | Delta(3,5)-Delta(2,4)-dienoyl-CoA isomerase, mitochondrial OS=Homo sapiens GN=ECH1 PE=1 SV=2 - [ECH1_HUMAN] | 60.97 | 13.72 | 1 | 5 | 5 | 7 | 328 | 35.8 | 8.00 |
| P48556 | 26S proteasome non-ATPase regulatory subunit 8 OS=Homo sapiens GN=PSMD8 PE=1 SV=2 - [PSMD8_HUMAN] | 51.83 | 12.00 | 1 | 5 | 5 | 5 | 350 | 39.6 | 9.70 |
| P11142 | Heat shock cognate 71 kDa protein OS=Homo sapiens GN=HSPA8 PE=1 SV=1 - [HSP7C_HUMAN] | 48.06 | 8.05 | 5 | 5 | 5 | 5 | 646 | 70.9 | 5.52 |
| P39687 | Acidic leucine-rich nuclear phosphoprotein 32 family member A OS=Homo sapiens GN=ANP32A PE=1 SV=1 - [AN32A_HUMAN] | 45.80 | 11.24 | 3 | 2 | 5 | 6 | 249 | 28.6 | 4.09 |
| Q92688 | Acidic leucine-rich nuclear phosphoprotein 32 family member B OS=Homo sapiens GN=ANP32B PE=1 SV=1 - [AN32B_HUMAN] | 45.52 | 9.96 | 5 | 2 | 5 | 6 | 251 | 28.8 | 4.06 |
| Q9NUI1 | Peroxisomal 2,4-dienoyl-CoA reductase OS=Homo sapiens GN=DECR2 PE=1 SV=1 - [DECR2_HUMAN] | 91.40 | 13.01 | 1 | 4 | 4 | 4 | 292 | 30.8 | 9.22 |
| Q8WXX5 | DnaJ homolog subfamily C member 9 OS=Homo sapiens GN=DNAJC9 PE=1 SV=1 - [DNJC9_HUMAN] | 86.52 | 15.00 | 1 | 4 | 4 | 4 | 260 | 29.9 | 5.73 |
| Q9UBR2 | Cathepsin Z OS=Homo sapiens GN=CTSZ PE=1 SV=1 - [CATZ_HUMAN] | 82.43 | 10.56 | 1 | 4 | 4 | 4 | 303 | 33.8 | 7.11 |
| P19105 | Myosin regulatory light chain 12A OS=Homo sapiens GN=MYL12A PE=1 SV=2 - [ML12A_HUMAN] | 78.02 | 24.56 | 3 | 4 | 4 | 5 | 171 | 19.8 | 4.81 |
| P06733 | Alpha-enolase OS=Homo sapiens GN=ENO1 PE=1 SV=2 - [ENOA_HUMAN] | 77.07 | 8.06 | 1 | 4 | 4 | 5 | 434 | 47.1 | 7.39 |
| P36543 | V-type proton ATPase subunit E 1 OS=Homo sapiens GN=ATP6V1E1 PE=1 SV=1 - [VATE1_HUMAN] | 72.06 | 13.27 | 2 | 4 | 4 | 4 | 226 | 26.1 | 8.00 |
| O43396 | Thioredoxin-like protein 1 OS=Homo sapiens GN=TXNL1 PE=1 SV=3 - [TXNL1_HUMAN] | 71.39 | 14.53 | 1 | 4 | 4 | 5 | 289 | 32.2 | 4.96 |
